# Account of Two Cases of Division of the Symphysis Pubis, Performed by Dr. Manchini, of Naples

**Published:** 1823-12

**Authors:** Richard Harrison


ART. IV.—*Account of two Cases of Division of the Symphysis Pubis, performed by* Dr. MANCHINI, *of Naples.* Communicated in a Letter to the Editors, from RICHARD HARRISON, M.D. &c.

THE following information may not be uninteresting to the readers of your Journal.—My friend, Dr. Manchini, professor of anatomy at Naples, has recently divided the symphysis pubis in two cases, in which it had been determined, by previous consultations, that the delivery could not be effected by the natural process. In both instances the children were born alive, and did well, and the mothers recovered. After the operation, which consisted in a simple division of the symphysis, the patients were put into warm baths, and the further separation of the bones and dilatation of the passages left to the efforts of nature. I know not exactly what time was necessary for this purpose in the first case; but, in the second, the delivery was accomplished eight hours after the operation, when the divided bones were found to have separated an inch and a half from each other. The parietal bones of the child's head overlapped each other very much, and the whole cranium was brought into the form of a cone, from the pressure it had sustained in effecting a passage through the openings, which were still narrow. This deformity afterwards disappeared, and both mother and child did well in every respect.

In the first case, no re-union of the divided parts took place, owing to their not having been brought into apposition after the delivery; from which circumstance, the power of walking has not been perfectly recovered, but is performed in a *straddling* manner. In the second case, the parts were brought together after the accouchement, and retained in their natural situation by means of rollers properly applied: the bones united, and no inconvenience of any kind was afterwards experienced. These cases were communicated to me by Dr. Crawford, who was present at the last of the two operations above alluded to.

*Argyll-street; November* 8*th.*

---

